# Knowledge and Anxiety about COVID-19 in the State of Qatar, and the Middle East and North Africa Region—A Cross Sectional Study

**DOI:** 10.3390/ijerph18126439

**Published:** 2021-06-14

**Authors:** Sathyanarayanan Doraiswamy, Sohaila Cheema, Patrick Maisonneuve, Amit Abraham, Ingmar Weber, Jisun An, Albert B. Lowenfels, Ravinder Mamtani

**Affiliations:** 1 Institute for Population Health, Weill Cornell Medicine—Qatar, Ar-Rayyan, Qatar; ama2006@qatar-med.cornell.edu (A.A.); ram2026@qatar-med.cornell.edu (R.M.); 2 Division of Epidemiology and Biostatistics, IEO European Institute of Oncology IRCCS, 20141 Milan, Italy; patrick.maisonneuve@ieo.it; 3 Qatar Computing Research Institute, Hamad Bin Khalifa University, Ar-Rayyan, Qatar; iweber@hbku.edu.qa (I.W.); an.jisun.221@gmail.com (J.A.); 4 Department of Surgery, New York Medical College, Valhalla, New York, NY 10595, USA; al_lowenfels@nymc.edu; 5 Department of Family Medicine, New York Medical College, Valhalla, New York, NY 10595, USA

**Keywords:** COVID-19, health information, misinformation, anxiety, knowledge

## Abstract

While the coronavirus disease 2019 (COVID-19) pandemic wreaked havoc across the globe, we have witnessed substantial mis- and disinformation regarding various aspects of the disease. We conducted a cross-sectional study using a self-administered questionnaire for the general public (recruited via social media) and healthcare workers (recruited via email) from the State of Qatar, and the Middle East and North Africa region to understand the knowledge of and anxiety levels around COVID-19 (April–June 2020) during the early stage of the pandemic. The final dataset used for the analysis comprised of 1658 questionnaires (53.0% of 3129 received questionnaires; 1337 [80.6%] from the general public survey and 321 [19.4%] from the healthcare survey). Knowledge about COVID-19 was significantly different across the two survey populations, with a much higher proportion of healthcare workers possessing better COVID-19 knowledge than the general public (62.9% vs. 30.0%, *p* < 0.0001). A reverse effect was observed for anxiety, with a higher proportion of very anxious (or really frightened) respondents among the general public compared to healthcare workers (27.5% vs. 11.5%, *p* < 0.0001). A higher proportion of the general public tended to overestimate their chance of dying if they become ill with COVID-19, with 251 (18.7%) reporting the chance of dying (once COVID-19 positive) to be ≥25% versus 19 (5.9%) of healthcare workers (*p* < 0.0001). Good knowledge about COVID-19 was associated with low levels of anxiety. Panic and unfounded anxiety, as well as casual and carefree attitudes, can propel risk taking and mistake-making, thereby increasing vulnerability. It is important that governments, public health agencies, healthcare workers, and civil society organizations keep themselves updated regarding scientific developments and that they relay messages to the community in an honest, transparent, unbiased, and timely manner.

## 1. Introduction

The coronavirus disease 2019 (COVID-19) pandemic has wreaked havoc across the globe and caused tremendous suffering and loss of human life. Countries around the world have responded by implementing public health measures (e.g., movement restrictions, lockdowns, facemask use, regular hand washing) to limit the virus’ spread. Despite these measures, several countries have struggled to contain the virus’ spread [1,2]. With the development and deployment of vaccines, the prospects of stemming the transmission of COVID-19 have brightened considerably, but this has since been complicated by the arrival of new variants of unknown virulence and possibly higher transmissibility [3,4].

In parallel to the pandemic, there remain questions and confusion regarding various aspects of the COVID-19 disease. Examples of these include modes of transmission, symptoms, management, and the availability, efficacy, and effectiveness of available vaccines. This has led to the dissemination of misinformation and disinformation; the former being false information that is unintentionally spread, and the latter being information spread with the intent to deceive [5]. Mis- and disinformation have contributed to civil unrest and protests against governmental measures implemented to curb the spread of the virus and have facilitated the spread of conspiracy theories and prejudice [6]. This phenomenon is not unique to COVID-19, as previous outbreaks led to similar ‘infodemics’, such as during the SARS outbreak in 2002–2003, the swine flu pandemic in 2009, and the Ebola outbreaks over the last decade [7,8]. Mis- and disinformation can negatively affect adherence to public health precautions, such as physical distancing and facemask use, contributing to rapid spread of the SARS-CoV-2 virus. [9]. It is critically important that communities get correct and timely information, particularly during the early stages of a pandemic. Controlling the spread of disease while also avoiding unnecessary panic is essential in any outbreak, and so it is important that we learn lessons from COVID-19 for future similar events.

It is also crucial that we determine the extent of healthcare workers’ knowledge of COVID-19. Healthcare workers have an increased risk of contracting COVID-19 and suffering severe disease due to high rates of exposure, a shortage of personal protective equipment, and inadequate infection and control practices [10]. A well-informed healthcare worker becomes a trustworthy source for communities to learn from [11]. Thus, we should prioritize gauging the knowledge and perceptions of healthcare workers as outbreaks unfold.

Qatar, a small country in the Middle East, was at high risk of being severely affected by the pandemic during the early stages due to its status as a major travel hub and since a large proportion of its population are highly mobile expatriates. The first case of COVID-19 was confirmed in Qatar on 27 February 2020. Thereafter, several public health measures were implemented to contain the virus’ spread within the community [12]. As of 28 March 2021, the country had reported 177,774 positive cases and 253 deaths [13]. We were interested in conducting this survey at the early stages of the pandemic to determine the baseline knowledge and perceptions towards COVID-19 among healthcare workers in Qatar and the general public in the wider Middle East and North Africa (MENA) region. This survey will help us to understand knowledge and anxiety levels at the early stage of the pandemic and support more robust healthcare communication in the early stages of future outbreaks.

## 2. Methods

### 2.1. Design

The study followed a cross-sectional design using a descriptive approach. Basic demographic characteristics were collected, together with knowledge, anxiety, and perceptions about SARS-CoV-2 and COVID-19.

### 2.2. Study Respondents and Eligibility Criteria

Convenience sampling methodology was used to recruit respondents. Adults aged 18 years or above, resident in a country in the MENA region, and who were fluent in English were invited to participate in the survey. Adults who were unable to consent, those who did not understand English, as well as those aged under 18 years were not eligible to participate.

### 2.3. Study Instrument

An English-language, self-administered, 12-item questionnaire was designed based on existing information available in the published literature or at the World Health Organization website https://www.who.int/emergencies/diseases/novel-coronavirus-2019/question-and-answers-hub (accessed on 12 March 2020). The questionnaire (Supplementary Material S1) included sections describing socio-demographic characteristics and knowledge and perceptions of COVID-19, such as modes of transmission, symptoms, and treatment. The questionnaire was anonymous, voluntary, and no personally identifiable information was collected. The questionnaire was piloted in a small sample of 10 individuals (general public, study faculty and staff, healthcare practitioners) and improved based on feedback. The questionnaire took about 5 min to complete on average. The questionnaires filled during the pilot were not used in the final analysis.

### 2.4. Recruitment

The study consisted of two recruitment strategies. First, the questionnaire link (Google Form) was distributed among approximately 3000 healthcare workers based in Qatar using the mailing list of Weill Cornell Medicine-Qatar’s Institute for Population Health. The questionnaire link was also advertised on Facebook and LinkedIn, which allowed us to recruit mainly from the general public in the Middle East. This allowed us to compare the knowledge base of healthcare workers to the general public. We estimated that the response from 420 healthcare workers would be representative of the 42,000 health professionals in Qatar, using a margin of error of 5% and 95% confidence interval levels, and 10% invalid questionnaires. We did not calculate a sample size for the general public survey but aimed to get a maximum number of completed questionnaires as possible within the recruitment timeline. The recruitment for this study took place from 01 April to 12 June 2020.

### 2.5. COVID-19 Knowledge and Anxiety

COVID-19 knowledge measure was derived from a score based on responses to 18 single- and multiple-choice questions about COVID-19 modes of transmission, symptoms, prevention, treatments, and outcomes. The knowledge score was constructed by adding one point for each correct answer and subtracting one point for each incorrect answer (as an incorrect answer signifies misconception and can propel risky behavior). The list of questions, answers, and points attribution is available in Supplementary Table S1. The total score ranging from −5 to +23 (23 corresponding to correct answers to all the questions) was categorized in three groups of knowledge: low (score of −5 to 12), average (score of 13 to 17), and good (score of 18 to 23).

Anxiety about COVID-19 was derived from responses to a single question in which respondents were asked whether they were anxious about contracting COVID-19. For anxiety, respondents were categorized in three groups: not/slightly anxious, anxious, very anxious/really frightened.

### 2.6. Statistical Analysis

The data collected through Google Forms were downloaded directly in Microsoft Excel format. Separate databases were created for data from the healthcare worker and general public surveys. The data from respective databases were then imported into SAS software version 9.4 [14] for further analysis. Distribution of respondents’ characteristics and response to the COVID-19-related questions in the general public and the healthcare workers questionnaire were compared using the chi-square test for categorical variables and the Mantel-Haenszel test for trend for ordinal variables. The Mantel-Haenszel test for trend was also used to test for the association between respondents’ characteristics (and their responses to selected COVID-19-related questions) and COVID-19 knowledge and anxiety about COVID-19. Bar graphs were built to facilitate the interpretation of the data, while full contingency tables were constructed and are provided in Supplementary Material. Multiple regression analysis using stepwise variable selection was used to identify independent predictors of knowledge and anxiety. Selected variables included the type of survey, sex, age, nationality, education, and activity. Only variables that met 0.15 significance level were selected in the multivariable model. Partial R-square and total model r-square were calculated to quantify the percent of the variation in the response that can be explained by single predictors and by all the predictors. *p*-values were two-sided and *p* < 0.05 were considered statistically significant.

### 2.7. Ethical Declaration

The Weill Cornell Medicine-Qatar Institutional Review Board (IRB) reviewed the proposal and determined that the current study was exempt from Qatari and United States (US) human subjects’ protection regulations and therefore did not require review by an IRB (IRB number 20-00007).

## 3. Results

A total of 3129 completed questionnaires on Google Forms (2709 from the general public survey and 420 from the healthcare workers survey) were downloaded into a Microsoft Excel database and exclusions performed stepwise. Questionnaires from respondents who reported that they had not heard about the coronavirus/COVID-19 disease outbreak (187 [6%]) were excluded from the analysis. Excluded from our analysis were also questionnaires (704 [22.5%]) in which a response to this question was found to be missing. Additionally, 295 (9.4%) poorly completed questionnaires with 10 or more missing answers and 285 (9.1%) social media questionnaires from people living outside the MENA region or with missing information about their country of residence were also excluded. The final dataset used for the analysis comprised of 1658 questionnaires (53.0% of received questionnaires; 1337 [80.6%] from the general public survey and 321 [19.4%] from the healthcare workers survey).

The characteristics of the survey respondents are provided in Table 1. The median age of respondents was 39 years (interquartile range, 30–51), and 862 (52.0%) were males. About half (*n* = 949, 57.2%) were nationals from a MENA country and about half (n = 672, 40.5%) were residing in a Gulf Cooperation Council (GCC) country, i.e., Bahrain, Kuwait, Oman, Saudi Arabia, Qatar, and the United Arab Emirates. The majority (*n* = 928, 56.0%) had completed an undergraduate degree. Respondents’ characteristics were significantly different in the two surveys, with a higher proportion of females, of people in their 30s, of non-MENA country nationality, and with post-graduate qualifications in the healthcare workers survey than in the general public survey (all *p*-values < 0.0001).

Responses to COVID-19-related questions in the two surveys are presented in Table 2. Nearly all respondents (*n* = 1584, 95.5%) considered themselves being knowledgeable of the risk of Coronavirus/COVID-19, with no difference across surveys. A slightly higher proportion of respondents to the healthcare workers survey heard about COVID-19 from the radio or TV and fewer in this group heard it from social media (Facebook, Twitter, etc.) than respondents to the general public survey (*p* = 0.02 and *p* < 0.0001 respectively). COVID-19 knowledge was evaluated from responses to a series of questions presented in Supplementary Table S1, with respondents ranked in three groups of about similar size. Knowledge was significantly different across surveys with a much higher proportion of respondents with good knowledge in the healthcare workers survey than in the general public survey (62.9% vs. 30.0%, *p* < 0.0001). Only 28 (8.7%) respondents to the healthcare workers survey had a poor knowledge against 477 (35.7%) in the general public survey. A reverse effect was observed for anxiety, with a higher proportion of very anxious (or really frightened) respondents in the general public survey compared to the healthcare workers survey (*p* < 0.0001). In general, a higher proportion of the general public worried about getting the disease (*p* = 0.004), being quarantined (*p* = 0.04), or dying from COVID-19 (*p* < 0.0001) than healthcare workers, while fear of transmitting COVID-19 to family or others was more common among healthcare workers (*p* < 0.0001). Finally, a higher proportion of the general public overestimated the chances of dying if they become ill with COVID-19, with 251 (18.7%) reporting a chance of dying of ≥25% versus 19 (5.9%) of healthcare workers (*p* < 0.0001).

**Figure 1:**
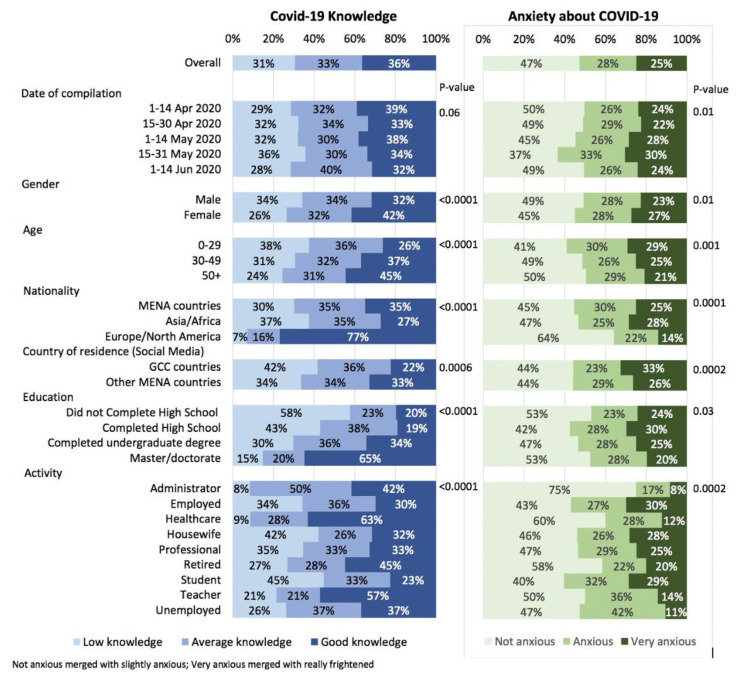
Association between participants’ characteristics, COVID-19, knowledge and anxiety about COVID-19 in 1658 responders to the survey.

Associations between respondents’ characteristics and COVID-19 knowledge and anxiety about COVID-19 are shown in Figure 1 and Supplementary Table S2. COVID-19 knowledge was higher in females than males, in older respondents, in nationals from Europe or North America, in more educated respondents, and in healthcare workers and teachers (all *p*-values < 0.0001). Females (*p* = 0.01), younger respondents (*p* = 0.001), respondents from Asia or Africa (*p* < 0.0001), residents in GCC countries, somewhat less educated respondents (completed high school, *p* = 0.03), employed persons, homemakers, and students reported higher levels of anxiety.

**Figure 2:**
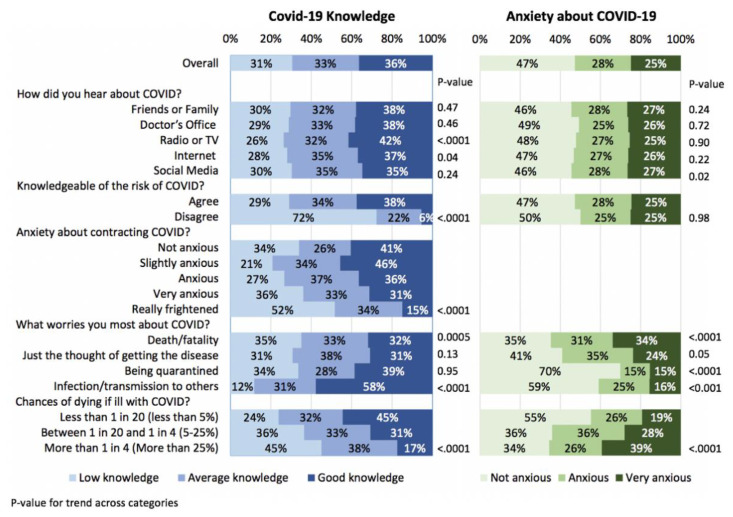
Association between participants’ answers to COVID-19 related questions, COVID-19 knowledge and anxiety about COVID-19 in 1658 responders to the survey.

The association between respondents’ answers to COVID-19-related questions and COVID-19 knowledge and anxiety about COVID-19 are shown in Figure 2 and Supplementary Table S3. Most of the respondents (72%) who reported that they were not knowledgeable of the risk of COVID-19 had indeed poor knowledge about the disease. Of relevance, a significant inverse association was found between knowledge and anxiety about COVID-19 (*p* < 0.0001), and, as a result, most factors associated with good knowledge were associated with less anxiety. For example, respondents who worried about death had lower knowledge (*p* = 0.0005) and more anxiety (*p* < 0.0001) while those who worried about infection/transmission to others had more knowledge (*p* < 0.0001) and less anxiety (*p* < 0.0001). Overestimation of the chance of dying if ill with COVID-19 was associated with low knowledge and increased anxiety (*p* < 0.0001).

In multivariable analysis (Table 3), the type of survey, sex, age, nationality, and education were all associated with COVID-19 knowledge. There was a wide variation in the level of knowledge among citizens from different regions. Respondents in the general public survey had poorer knowledge, whereas female and older respondents had better knowledge. Finally, an increasing level of education was associated with increasing knowledge. Education is the strongest predictor of knowledge (R-square = 0.098). Overall, these five variables explain about 20% of COVID-9 knowledge (Model R-square = 0.195).

Period of completion, type of survey, sex, and nationality were associated with anxiety about COVID-19. Anxiety increased from April to May-June 2020. Respondents to the social media survey and female respondents were more anxious. Citizens from Europe and North America were less anxious than citizens from MENA countries who completed the survey, Asia or Africa. Overall, these three variables explain very little anxiety (model R-square = 0.044).

## 4. Discussion

We learned several key lessons from this study. Firstly, we were able to establish that the knowledge of healthcare workers about COVID-19 was good during the early stages of the pandemic in Qatar, a phenomenon likely to be shared across the wider MENA region. The growing demand for personal protective equipment (PPE) by frontline healthcare workers worldwide (understanding the infectiousness of the disease and gathering evidence on the type of PPEs required) during the early stages of the pandemic is a testimony to this [15]. It was also notable that most healthcare workers, though slightly anxious, were not frightened about COVID-19 despite its novelty and unknowns. This is an important aspect in outbreak control, as first responders should be aware of the risks posed by a disease and precautions required and should neither panic nor be pre-occupied with fear [16]. Without the consistent efforts of frontline healthcare workers during the COVID-19 pandemic, the world would not be what it is now, a year since the start of the pandemic. The healthcare worker lives that were lost in the early stages of the pandemic in many countries were, in many instances, due to the non-availability of timely personal protective equipment rather than negligence on their part [17].

The findings from our general public survey are a stark contrast to the healthcare worker survey in the levels of knowledge (62.9% vs. 30.0%) and anxiety (27.5% vs. 11.5%) demonstrated. It is not surprising that those who responded to our general public survey relied mainly on social media for information about the pandemic. Social media as a double-edged sword has been well documented even before the pandemic and this has been substantiated further over the past year [18]. Uncontrolled media in general and social media in particular risk rapid spread of myths and misconceptions, which was clearly evident with the myths surrounding the cure for COVID-19 with chloroquine, bleach, etc. [19]. Mis- and disinformation, if not recognized and tackled early, will jeopardize outbreak control efforts and cost lives in the short-, medium-, and long-term [20].

We found a statistically significant negative association between knowledge and anxiety levels in our survey. Anxiety can compromise the mental health of individuals and make them indulge in risky behaviors that could disproportionally affect their overall health and well-being [21]. A case in point is when patients with chronic medical conditions chose to seek emergency medical care in hospitals and others who went on to self-imposed strict quarantine and social isolation when restricted movement, physical distancing, and facemask use would have been sufficient [22].We compared the results of our study with several other reports from countries in Europe [23], the Caribbean region [24], the United States [25], China [26] and a summary report from 35 different countries [27]. In general, the levels of anxiety we found in our study were similar to levels of anxiety about Covid-19 in these selected reports. As in our report, older patients reported less severe levels of anxiety or stress. However, unlike our report, other reports found females had higher anxiety levels than males. Predictably, there are other country-specific variables which we did not measure, such as government activities and newspaper reports that have an important impact on population responses to the COVID-19 pandemic [28].

Overestimating the chances of dying and the fear of quarantining were standout reasons for anxiety among the general public. Fear of transmitting the disease to others including patients and family members dominated the minds of healthcare workers. The altruistic nature of the healthcare workers of putting others first could possibly explain the fear of transmission to others rather than the risk for their lives [29]. It is interesting to note that those with greater knowledge levels were also more socially responsible as reflected by their greater concern of transmitting the disease to others. It is also possible that those whose natural tendency to behave in a socially responsible manner would have sought knowledge and from the right sources to practice evidence-based behaviors proportionate to the threat posed by the disease [30].

It is not entirely surprising that people with more education, those from wealthier nations with more advanced health systems, healthcare workers, and teachers had higher levels of knowledge on COVID-19. However, it is interesting to note that older people had higher knowledge levels than younger people. Older people, who were one of the most vulnerable groups, were better informed, and this success may have been due to targeted messaging to older people based on data that was emerging from countries worst affected during the early stages of the pandemic. This finding is similar to other studies [31,32].

Our study also found high COVID-19-related anxiety levels among students, employed persons, and homemakers. This may be related to their perceived inability to continue with their respective roles (missing out on school [33]/loss of job if absent from work [34]/inability to tend to children and accomplish home tasks [35]) if diagnosed with COVID-19, rather than the fear of the direct consequences of the disease itself. It was interesting to note that women, though more knowledgeable than men, were likely to feel more anxious about acquiring the disease. This would be an area for future qualitative research as COVID-19’s impact on the genders continues to be better understood [36].

Our study is the first of its kind in the MENA region to look for an association between knowledge and anxiety levels. Our cross-sectional study, with a large sample, was able to obtain the perspectives of both healthcare workers and the general public in a simultaneous survey. Though the diversity is rich in the sample, we acknowledge the low response rate in the healthcare worker survey. While the chances of convergence between the two groups (healthcare workers and general public recruited through social media) was minimal, it is possible that a small proportion of healthcare workers responded to the social media survey. Duplication (responses by the same individuals in both surveys) cannot also be ruled out as both surveys were anonymous. Much more needs to be learned about responding to this and other pandemics because our multivariable models reveal that there must be other explanatory variables which were not captured in this survey.

## 5. Conclusions

The COVID-19 pandemic continues to teach important public health lessons to the world. Communities empowered with the correct knowledge make evidence-based decisions to protect themselves and their communities. Panic and unfounded anxiety, as well as a casual and carefree attitude, both can increase risk-taking and mistake-making, thereby increasing vulnerability to infection. It is important that governments, public health agencies, healthcare workers, and civil society organizations keep themselves updated on the scientific developments and transmit these messages to their communities in an honest, transparent, unbiased, and timely manner. Health institutions and stakeholders should also keep healthcare workers updated with evolving information and protective measures. Unaddressed anxiety among healthcare workers can undermine the public health response to the pandemic. There also remain several unknowns as to why some people react the way they do in a crisis such as COVID-19. We believe there is more scope for exploratory research to understand human behaviors and the drivers of their actions during a pandemic. They can guide us in the development of a robust and proactive approach in battling future outbreaks and pandemics.

## Figures and Tables

**Table 1:** Characteristics of respondents to the general public and healthcare workers surveys.

	All	General Public Survey	Healthcare Workers Survey	*p*-Value
**All respondents**	1658 (100.0)	1337 (100.0)	321 (100.0)	
**Date of compilation**				
1–14 Apr 2020	757 (45.7)	601 (45.0)	156 (48.6)	
15–30 Apr 2020	335 (20.2)	270 (20.2)	65 (20.2)
1–14 May 2020	181 (10.9)	152 (11.4)	29 (9.0)	
15–31 May 2020	265 (16.0)	238 (17.8)	27 (8.4)	
1–14 Jun 2020	95 (5.7)	76 (5.7)	19 (5.9)	0.004
Missing	25 (1.5)	0 (0.0)	25 (7.8)	
**Gender**				
Male	862 (52.0)	725 (54.2)	137 (42.7)	
Female	792 (47.8)	608 (45.5)	184 (57.3)	0.0002
**Age**				
0–29 years	407 (24.5)	358 (26.8)	49 (15.3)	
30–49 years	746 (45.0)	572 (42.8)	174 (54.2)	
50+ years	483 (29.1)	395 (29.5)	88 (27.4)	<0.0001
**Nationality**				
MENA countries	949 (57.2)	843 (63.1)	106 (33.0)	
Other countries from Asia/Africa	505 (30.5)	368 (27.5)	137 (42.7)	
Europe/North America	143 (8.6)	88 (6.6)	55 (17.1)	
Oceania/South America	17 (1.0)	8 (0.6)	9 (2.8)	<0.0001
**Country of residence from general public survey**				
GCC countries	672 40.5)	351 (26.3)	321 (100.0)	
Other countries from the MENA region	986 (59.5)	986 (73.7)		<0.0001
**Education**				
Did not Complete High School	68 (4.1)	58 (4.3)	10 (3.1)	
Completed High School	311 (18.8)	303 (22.7)	8 (2.5)	
Completed undergraduate degree	928 (56.0)	812 (60.7)	116 (36.1)	
Master/doctorate	318 (19.2)	140 (10.5)	178 (55.5)	<0.0001
**You are best described as**				
Administrator	12 (0.7)	0 (0.0)	12 (3.7)	
Employed	750 (45.2)	750 (56.1)	0 (0.0)	
Healthcare	247 (14.9)	10 (0.7)	237 (73.8)	
Housewife	39 (2.4)	39 (2.9)	0 (0.0)	
Professional	209 (12.6)	209 (15.6)	0 (0.0)	
Retired	67 (4.0)	67 (5.0)	0 (0.0)	
Student	191 (11.5)	191 (14.3)	0 (0.0)	
Teacher	28 (1.7)	13 (1.0)	15 (4.7)	<0.0001

Missing values: gender (*n* = 4), age (*n* = 22), marital status (*n* = 23), nationality (*n* = 44), education (*n* = 33), activity (*n* = 96).

**Table 2:** Distribution of COVID-19-related responses across surveys.

	All	General Public Survey	Healthcare Workers Survey	*p*-Value
**All respondents**	1658 (100.0)	1337 (100.0)	321 (100.0)	
**I am knowledgeable of the risk of Coronavirus/ COVID-19**				
Agree	1584 (95.5)	1270 (95.0)	314 (97.8)	
Disagree	36 (2.2)	33 (0.2)	3 (0.9)	0.09
**How did you hear about Coronavirus/COVID-19?**				
Friends or Family	535 (32.3)	436 (32.6)	99 (30.8)	0.53
Doctor’s Office	274 (16.5)	210 (15.7)	64 (19.9)	0.07
Radio or TV	951 (57.4)	748 (55.9)	203 (63.2)	0.02
Internet	1142 (68.9)	923 (69.0)	219 (68.2)	0.74
Social Media (Facebook, Twitter etc.)	1127 (68.0)	954 (71.4)	173 (53.9)	<0.0001
**Knowledge about Coronavirus/COVID-19**				
Low (score −5 to 12)	505 (30.5)	477 (35.7)	28 (8.7)	
Average (score 13 to 17)	547 (33.0)	456 (34.1)	91 (28.3)	
Good (score 18 to 23)	603 (36.4)	401 (30.0)	202 (62.9)	<0.0001
**Are you anxious about contracting Coronavirus/COVID-19?**				
Not or slightly anxious	255 (15.4)	196 (14.7)	59 (18.4)	
Slightly anxious	516 (31.1)	384 (28.7)	132 (41.1)	
Anxious	456 (27.5)	366 (27.4)	90 (28.0)	
Very anxious	211 (12.7)	181 (13.5)	30 (9.3)	
Really frightened	194 (11.7)	187 (14.0)	7 (2.2)	<0.0001
**What worries you most about Coronavirus/COVID-19?**				
Just the thought of getting the disease	418 (25.2)	360 (26.9)	58 (18.1)	0.004
Being quarantined	158 (9.5)	138 (10.3)	20 (6.2)	0.04
Transmitting to family/others	154 (9.3)	48 (3.6)	106 (33.0)	<0.0001
Death/fatality	651 (39.3)	561 (42.0)	90 (28.0)	<0.0001
**If you become ill with Coronavirus/COVID-19, the chances of dying are:**				
Less than 1 in 20 (less than 5%)	990 (59.7)	751 (56.2)	239 (74.5)	
Between 1 in 20 and 1 in 4 (5–25%)	342 (20.6)	287 (21.5)	55 (17.1)	
Between 1 in 4 and 1 in 2 (25–50%)	129 (7.8)	114 (8.5)	15 (4.7)	
More than 1 in 2 (More than 50%)	141 (8.5)	137 (10.2)	4 (1.2)	<0.0001

**Table 3:** Multivariable analysis of demographic factors associated with knowledge and anxiety.

	COVID-19 Knowledge				Anxiety about COVID-19			
	Parameter Estimate	Standard Error	*p*-Value	Partial R-Square	Parameter Estimate	Standard Error	*p*-Value	Partial R-Square
**Date of compilation**								
April					Ref			0.004
May–June					0.15	0.06	0.02	
**Survey**								
Healthcare survey	Ref			0.031	Ref			0.024
Social media survey	−2.81	0.36	<0.0001		0.43	0.08	<0.0001	
**Gender**				0.009				0.007
Male	Ref				Ref			
Female	1.13	0.25	<0.0001		0.22	0.06	0.0004	
**Age**				0.007				
0–29	Ref							
30–49	0.34	0.32	0.29					
50+	1.15	0.36	0.001					
**Nationality**				0.051				
MENA countries	Ref				Ref			0.008
Asia/Africa	−1.60	0.29	<0.0001		0.06	0.07	0.42	
Europe/North America	2.58	0.47	<0.0001		−0.36	0.11	0.001	
**Education**				0.098				
Did not Complete High School	Ref							
Completed High School	1.98	0.68	0.003					
Completed undergraduate degree	3.71	0.64	<0.0001					
Master/doctorate	4.88	0.70	<0.0001					
**Model R-square**				0.195				0.044

No other variables met the 0.15 significance level for entry into the model.

## Data Availability

The data presented in this study are available on request from the corresponding author. The data are not publicly available due to privacy restrictions.

## Supplementary Materials

The following are available online at https://www.mdpi.com/article/10.3390/ijerph18126439/s1, Material 1: Questionnaire, Table S1: Questions about knowledge of coronavirus and elaboration of the COVID-19 knowledge score., Table S2: Association between respondents’ characteristics, COVID-19 knowledge, and anxiety about COVID-19. Table S3: Association between respondents’ answers to COVID-19 related questions, COVID-19 knowledge and anxiety about COVID-19.
