# Supplementary material for: Knowledge and Anxiety about COVID-19 in the State of Qatar, and the Middle East and North Africa Region—A Cross Sectional Study
Source: Int J Environ Res Public Health. 2021 Jun 14;18(12):6439. doi: 10.3390/ijerph18126439 (PMC8296266; doi:10.3390/ijerph18126439)
Supplement: Supplementary file 1 [file ijerph-18-06439-s001.zip › ijerph-1216607-SI.pdf]

**Supplementary Material 1: Questionnaire**

**Front Sheet (Healthcare workers)**

Today's date: (DD/MM/YY): ___________

Gender: ☐ Male
☐ Female

Age: ________

Nationality: ______________

Marital status:
- ☐ Single
- ☐ Married
- Other:_______

Your highest qualification:
- ☐ Bachelors
- ☐ Masters
- ☐ Doctorate
- ☐ Other ___________

Your profession is:
- ☐ Physician
- ☐ Allied health practitioner
- ☐ Dentist
- ☐ Nurse
- ☐ Pharmacist
- ☐ Complementary Medicine
- ☐ Educator
- ☐ Administrator
- ☐ Other __________

Have you heard about the Coronavirus/COVID-19 disease outbreak?
- ☐ Yes
- ☐ No

**If you haven't heard about the Coronavirus/COVID-19 disease outbreak, you do not need to proceed. Please end the survey here.**

…………………………………………………………………………………………

If you have heard about the Coronavirus/COVID-19 disease outbreak, please answer the following questions:

**Front Sheet (General Public)**

Today's date: (DD/MM/YY): ___________

Gender: ☐ Male
☐ Female

Age: ________

Nationality: ______________

Country of Residence:

Marital status:
- ☐ Single
- ☐ Married
- Other: _______

Education:
- ☐ Did not Complete High School
- ☐ Completed High School
- ☐ Completed College degree
- ☐ Other ___________

You are best described as:
- ☐ Student
- ☐ Employed
- ☐ Self-employed
- ☐ Other __________

Have you heard about the Coronavirus/COVID-19 disease outbreak?
- ☐ Yes
- ☐ No

**If you haven't heard about the Coronavirus/COVID-19 disease outbreak, you do not need to proceed. Please end the survey here.**

……………………………………………………………………………………………

If you have heard about the Coronavirus/COVID-19 disease outbreak, please answer the following questions:

**Coronavirus/COVID-19 Questionnaire**

Q1) How did you hear about Coronavirus/COVID-19? **(Check all that apply)**

☐ Friends or Family
☐ Doctor's Office
☐ Radio or TV
☐ Internet
☐ Social Media (Facebook, Twitter etc.)
☐ Other source __________

Q2) How does Coronavirus/COVID-19 spread? **(Check all that apply)**

☐ Drinking polluted water
☐ Eating contaminated food
☐ Through coughing and sneezing (i.e., airborne)
☐ By touching contaminated surfaces (doors, knobs, clothing etc.)
☐ Close contact with an infected person
☐ By eating Chinese or Asian food
☐ By touching eyes, nose or mouth
☐ Other (please specify________________)

Q3) Who is at a higher risk of getting COVID-19? **(Check all that apply)**

☐ Adult men
☐ Adult women
☐ Pregnant women
☐ Children
☐ Elderly
☐ Other ________
☐ All of the above

Q4) If you have been exposed to Coronavirus/COVID-19, symptoms normally develop within:

☐ 24 hours
☐ 2-14 days
☐ Greater than 14 days
☐ Don't know

Q5) Which of the following measures have been proposed to reduce the risk of Coronavirus/COVID-19 transmission among the general population? **(Check all that apply)**

☐ Face masks
☐ Hand washing
☐ Avoiding crowded public places
☐ Frequent showers
☐ Drinking only bottled water

Q6) Is there a vaccine to prevent Coronavirus/COVID-19?

- ☐ Yes
- ☐ No
- ☐ I don't know

Q7) If you have been vaccinated against flu, will it help protect you from Coronavirus/COVID-19?

- ☐ Yes
- ☐ No
- ☐ I don't know

Q8) Have you ever suffered from flu?

- ☐ Yes
- ☐ No
- ☐ I don't know

Q9) For the following statements, please check if you agree/disagree/don't know

| | Agree | Disagree | Don't know |
|---|---|---|---|
| a. I am knowledgeable of the risk of Coronavirus/ COVID-19 | | | |
| b. The disease has only been diagnosed in China or in people who have been visiting/returning from China | | | |
| c. I can control my exposure to Coronavirus/ COVID-19 | | | |
| d. Pets (e.g. cat/dog) at home can spread the virus | | | |
| e. Eating certain foods like oranges, garlic can help protect me from Coronavirus/ COVID-19 | | | |
| f. The Coronavirus/ COVID-19 is thought to have originated from animals | | | |
| g. Early symptoms of Coronavirus/ COVID-19 are different from other respiratory diseases | | | |
| h. Coronavirus/ COVID-19 can be **treated** with antibiotics | | | |

| | | | |
|---|---|---|---|
| i. Coronavirus/ COVID-19 can be **prevented** with antibiotics | | | |
| j. Using a hand dryer effectively kills Coronavirus/COVID-19 | | | |
| k. Exposure to Coronavirus/ COVID-19 always leads to sickness | | | |
| l. If you contract Coronavirus/COVID-19, having diabetes or other chronic diseases, this increases the risk of death | | | |
| m. Older persons have a higher death risk after being infected with Coronavirus/COVID-19 | | | |
| n. Only people who have travelled to China or other Asian countries are at risk of developing Coronavirus/COVID-19 disease? | | | |
| o. It is safe to receive a letter or package from China or other Asian countries | | | |

Q10) Are you anxious about contracting Coronavirus/COVID-19?

- ☐ Not anxious
- ☐ Slightly anxious
- ☐ Anxious
- ☐ Very anxious
- ☐ Really frightened

Q11) What worries you most about Coronavirus/COVID-19?

- ☐ Death/ fatality
- ☐ Just the thought of getting the disease
- ☐ Being quarantined
- ☐ Infection in children
- ☐ Infection in older people
- ☐ Other ________
- ☐ None of the above

Q12) If you become ill with Coronavirus/COVID-19, the chances of dying are:

- ☐ Less than 1 in 20 (less than 5%)
- ☐ Between 1 in 20 and 1 in 4 (5-25%)
- ☐ Between 1 in 4 and 1 in 2 (25-50%)
- ☐ More than 1 in 2 (More than 50%)

**End of questionnaire. Thank you for answering these questions**

**Supplementary Table 1.** Questions about knowledge of coronavirus and elaboration of the COVID-19 knowledge score.

| **Questions about COVID-19** | | **SCORE points** | **All** | **General Public survey** | **Healthcare Workers survey** | **p-value** |
|---|---|---|---|---|---|---|
| | | **[-23 +23]** | **1658 (100.0)** | **1337 (100.0)** | **321 (100.0)** | |
| **Is there a vaccine to prevent Coronavirus/COVID-19?** | | | | | | |
| Yes | Wrong | -1 | 28 ( 1.7) | 26 ( 1.9) | 2 ( 0.6) | |
| No | Correct | +1 | 1473 (88.8) | 1163 (87.0) | 310 (96.6) | 0.10 |
| **If you have been vaccinated against flu, will it help protect you from Coronavirus/COVID-19?** | | | | | | |
| Yes | Wrong | -1 | 88 ( 5.3) | 77 ( 5.8) | 11 ( 3.4) | |
| No | Correct | +1 | 1195 (72.1) | 933 (69.8) | 262 (81.6) | 0.04 |
| **How does Coronavirus/COVID-19 spread?** | | | | | | |
| Drinking polluted water | Wrong | -1 | 78 ( 4.7) | 72 ( 5.4) | 6 ( 1.9) | 0.005 |
| Eating contaminated food | Wrong | -1 | 174 (10.5) | 152 (11.4) | 22 ( 6.9) | 0.02 |
| Through coughing and sneezing (i.e., airborne) | Correct | +1 | 1403 (84.6) | 1123 (84.0) | 280 (87.2) | 0.17 |
| By touching contaminated surfaces (doors, knobs, clothing etc.) | Correct | +1 | 1482 (89.4) | 1178 (88.1) | 304 (94.7) | 0.0004 |
| Close contact with an infected person | Correct | +1 | 1538 (92.8) | 1234 (92.3) | 304 (94.7) | 0.18 |
| By eating Chinese or Asian food | Wrong | -1 | 58 ( 3.5) | 55 ( 4.1) | 3 ( 0.9) | 0.004 |
| By touching eyes, nose or mouth | Correct | +1 | 1312 (79.1) | 1051 (78.6) | 261 (81.3) | 0.32 |
| **Which of the following measures have been proposed to reduce the risk of COVID-19 transmission among the general population?** | | | | | | |
| Face masks | Correct | +1 | 1389 (83.8) | 1116 (83.5) | 273 (85.0) | 0.56 |
| Hand washing | Correct | +1 | 1568 (94.6) | 1252 (93.6) | 316 (98.4) | 0.0003 |
| Avoiding crowded public places | Correct | +1 | 1584 (95.5) | 1267 (94.8) | 317 (98.8) | 0.001 |
| Frequent showers | Wrong | -1 | 317 (19.1) | 259 (19.4) | 58 (18.1) | 0.64 |
| Drinking only bottled water | Wrong | -1 | 113 ( 6.8) | 102 ( 7.6) | 11 ( 3.4) | 0.006 |
| **If you have been exposed to Coronavirus/COVID-19, symptoms normally develop within:** | | | | | | |
| 24 hours | Wrong | -1 | 26 ( 1.6) | 25 ( 1.9) | 1 ( 0.3) | |
| 2-14 days | Correct | +1 | 1344 (81.1) | 1053 (78.8) | 291 (90.7) | |
| Greater than 14 days | Wrong | -1 | 168 (10.1) | 148 (11.1) | 20 ( 6.2) | 0.001 |
| **For the following items, please rate your level of agreement with the statements** | | | | | | |
| **The disease has only been diagnosed in China or in people who have been visiting/returning from China** | | | | | | |
| Agree | Wrong | -1 | 327 (19.7) | 290 (21.7) | 37 (11.5) | |
| Disagree | Correct | +1 | 1239 (74.7) | 963 (72.0) | 276 (86.0) | <.0001 |
| **I can control my exposure to Coronavirus/COVID-19** | | | | | | |
| Agree | Correct | +1 | 1178 (71.0) | 921 (68.9) | 257 (80.1) | |
| Disagree | Wrong | -1 | 327 (19.7) | 281 (21.0) | 46 (14.3) | 0.002 |
| **Pets (e.g., cat/dog) at home can spread the virus** | | | | | | |
| Agree | Wrong | -1 | 383 (23.1) | 317 (23.7) | 66 (20.6) | |
| Disagree | Correct | +1 | 1004 (60.6) | 813 (60.8) | 191 (59.5) | 0.49 |
| **Eating certain foods like oranges, garlic can help protect me from Coronavirus/COVID-19** | | | | | | |
| Agree | Wrong | -1 | 644 (38.8) | 566 (42.3) | 78 (24.3) | |
| Disagree | Correct | +1 | 818 (49.3) | 616 (46.1) | 202 (62.9) | <.0001 |
| **The Coronavirus/COVID-19 is thought to have originated from animals** | | | | | | |
| Agree | Correct | +1 | 1134 (68.4) | 877 (65.6) | 257 (80.1) | |
| Disagree | Wrong | -1 | 317 (19.1) | 291 (21.8) | 26 ( 8.1) | <.0001 |
| **Coronavirus/COVID-19 can be treated with antibiotics** | | | | | | |
| Agree | Wrong | -1 | 206 (12.4) | 183 (13.7) | 23 ( 7.2) | |
| Disagree | Correct | +1 | 1275 (76.9) | 1000 (74.8) | 275 (85.7) | 0.0003 |
| **Coronavirus/ COVID-19 can be prevented with antibiotics** | | | | | | |
| Agree | Wrong | -1 | 133 (8.0) | 123 (9.2) | 10 ( 3.1) | |
| Disagree | Correct | +1 | 1337 (80.6) | 1050 (78.5) | 287 (89.4) | <.0001 |
| **Using a hand dryer effectively kills Coronavirus/COVID-19** | | | | | | |
| Agree | Wrong | -1 | 256 (15.4) | 235 (17.6) | 21 ( 6.5) | |
| Disagree | Correct | +1 | 1143 (68.9) | 892 (66.7) | 251 (78.2) | <.0001 |
| **Exposure to Coronavirus/COVID-19 always leads to sickness** | | | | | | |
| Agree | Wrong | -1 | 630 (38.0) | 575 (43.0) | 55 (17.1) | |

| | | | | | | |
|---|---|---|---|---|---|---|
| Disagree | Correct | +1 | 878 (53.0) | 634 (47.4) | 244 (76.0) | <.0001 |
| **If you contract Coronavirus/COVID-19 having diabetes or other chronic diseases, this increases the risk of death** | | | | | | |
| Agree | Correct | +1 | 1491 (89.9) | 1178 (88.1) | 313 (97.5) | |
| Disagree | Wrong | -1 | 112 ( 6.8) | 110 ( 8.2) | 2 ( 0.6) | <.0001 |
| **Older persons have a higher death risk after being infected with Coronavirus/COVID-19** | | | | | | |
| Agree | Correct | +1 | 1509 (91.0) | 1195 (89.4) | 314 (97.8) | |
| Disagree | Wrong | -1 | 107 ( 6.5) | 103 ( 7.7) | 4 ( 1.2) | <.0001 |
| **Only people who have travelled to China or other Asian countries are at risk of developing Coronavirus/COVID-19 disease?** | | | | | | |
| Agree | Wrong | -1 | 161 ( 9.7) | 141 (10.5) | 20 ( 6.2) | |
| Disagree | Correct | +1 | 1392 (84.0) | 1094 (81.8) | 298 (92.8) | 0.007 |
| **It is safe to receive a letter or package from China or other Asian countries** | | | | | | |
| Agree | Correct | +1 | 893 (53.9) | 677 (50.6) | 216 (67.3) | |
| Disagree | Wrong | -1 | 497 (30.0) | 442 (33.1) | 55 (17.1) | <.0001 |
| **Median Covid-19 knowledge score [range]** | points | | 16 [-5 to 23] | 15 [-5 to 23] | 19 [0 to 23] | <.0001 |

0 points were given to missing values

**Supplementary Table 2.** Association between respondents' characteristics, COVID-19 knowledge, and anxiety about COVID-19

| | **COVID-19 Knowledge** | | | | **Anxiety about COVID-19** | | | |
|---|---|---|---|---|---|---|---|---|
| | **Low (-5 to 12)** | **Average (13 to 17)** | **Good (18 to 23)** | **p-value trend** | **Not anxious** | **Anxious** | **Very anxious** | **p-value trend** |
| | 505 (100.0) | 547 (100.0) | 603 (100.0) | | 771 (100.0) | 456 (100.0) | 405 (100.0) | |
| **Date of compilation** | | | | | | | | |
| 1-14 Apr 2020 | 216 (42.8) | 245 (44.8) | 295 (48.9) | | | | | |
| 15-30 Apr 2020 | 108 (21.4) | 115 (21.0) | 112 (18.6) | | 370 (48.0) | 197 (43.2) | 178 (44.0) | |
| 1-14 May 2020 | 58 (11.5) | 54 ( 9.9) | 69 (11.4) | | 161 (20.9) | 94 (20.6) | 74 (18.3) | |
| 15-31 May 2020 | 94 (18.6) | 80 (14.6) | 89 (14.8) | | 81 (10.5) | 47 (10.3) | 51 (12.6) | |
| 1-14 Jun 2020 | 27 ( 5.3) | 38 ( 6.9) | 30 ( 5.0) | 0.06 | 96 (12.5) | 87 (19.1) | 80 (19.8) | 0.01 |
| **Gender** | | | | | 45 ( 5.8) | 24 ( 5.3) | 22 ( 5.4) | |
| Male | 294 (58.2) | 293 (53.6) | 273 (45.3) | | | | | |
| Female | 209 (41.4) | 253 (46.3) | 329 (54.6) | <.0001 | 417 (54.1) | 238 (52.2) | 192 (47.4) | 0.01 |
| **Age** | | | | | 351 (45.5) | 217 (47.6) | 213 (52.6) | |
| 0-29 | 152 (30.1) | 147 (26.9) | 106 (17.6) | | | | | |
| 30-49 | 228 (45.1) | 242 (44.2) | 275 (45.6) | | 164 (21.3) | 120 (26.3) | 117 (28.9) | |
| 50+ | 118 (23.4) | 150 (27.4) | 215 (35.7) | <.0001 | 357 (46.3) | 193 (42.3) | 184 (45.4) | 0.001 |
| **Nationality** | | | | | 240 (31.1) | 137 (30.0) | 99 (24.4) | |
| MENA countries | 286 (56.6) | 331 (60.5) | 331 (54.9) | | | | | |
| Asia/Africa | 188 (37.2) | 178 (32.5) | 137 (22.7) | | 418 (54.2) | 285 (62.5) | 234 (57.8) | |
| Europe/North America | 10 ( 2.0) | 23 ( 4.2) | 110 (18.2) | <.0001 | 231 (30.0) | 123 (27.0) | 141 (34.8) | 0.0001 |
| **Country of residence for Social Media Questionnaires** | | | | | 91 (11.8) | 31 ( 6.8) | 20 ( 4.9) | |
| GCC countries | 146 (28.9) | 126 (23.0) | 78 (12.9) | | | | | |
| Other countries from the MENA region | 331 (65.5) | 330 (60.3) | 323 (53.6) | 0.0006 | 152 (19.7) | 80 (17.5) | 112 (27.7) | 0.0002 |
| **Education** | | | | | 428 (55.5) | 286 (62.7) | 256 (63.2) | |
| Did not Complete High School | 38 ( 7.5) | 15 ( 2.7) | 13 ( 2.2) | | | | | |
| Completed High School | 133 (26.3) | 118 (21.6) | 59 ( 9.8) | | 35 ( 4.5) | 15 ( 3.3) | 16 ( 4.0) | |
| Completed undergraduate degree | 276 (54.7) | 334 (61.1) | 318 (52.7) | | 130 (16.9) | 85 (18.6) | 91 (22.5) | |
| Master/doctorate | 47 ( 9.3) | 65 (11.9) | 206 (34.2) | <.00001 | 425 (55.1) | 259 (56.8) | 227 (56.0) | 0.03 |
| **You are best described as** | | | | | 166 (21.5) | 88 (19.3) | 62 (15.3) | |
| Administrator | 1 ( 0.2) | 6 ( 1.1) | 5 ( 0.8) | | | | | |
| Employed | 257 (50.9) | 270 (49.4) | 223 (37.0) | | 9 ( 1.2) | 2 ( 0.4) | 1 ( 0.2) | |
| Healthcare | 21 ( 4.2) | 70 (12.8) | 156 (25.9) | | 318 (41.2) | 201 (44.1) | 221 (54.6) | |
| Housewife | 16 ( 3.2) | 10 ( 1.8) | 12 ( 2.0) | | 147 (19.1) | 68 (14.9) | 30 ( 7.4) | |
| Professional | 72 (14.3) | 68 (12.4) | 68 (11.3) | | 18 ( 2.3) | 10 ( 2.2) | 11 ( 2.7) | |
| Retired | 18 ( 3.6) | 19 ( 3.5) | 30 ( 5.0) | | 94 (12.2) | 58 (12.7) | 50 (12.3) | |
| Student | 85 (16.8) | 62 (11.3) | 43 ( 7.1) | | 38 ( 4.9) | 14 ( 3.1) | 13 ( 3.2) | |
| Teacher | 6 ( 1.2) | 6 ( 1.1) | 16 ( 2.7) | | 75 ( 9.7) | 60 (13.2) | 54 (13.3) | |
| Unemployed | 5 ( 1.0) | 7 ( 1.3) | 7 ( 1.2) | <.0001 | 14 ( 1.8) | 10 ( 2.2) | 4 ( 1.0) | 0.0002 |

Not anxious merged with slightly anxious; Very anxious merged with really frightened
Knowledge is missing for 3 respondents; Anxiety is missing for 26 respondents

**Supplementary Table 3.** Association between respondents' answers to COVID-19 related questions, COVID-19 knowledge and anxiety about COVID-19

| | **COVID-19 Knowledge** | | | | **Anxiety about COVID-19** | | | |
|---|---|---|---|---|---|---|---|---|
| | **Low (-5 to 12)** | **Average (13 to 17)** | **Good (18 to 23)** | **p-value trend** | **Not anxious** | **Anxious** | **Very anxious** | **p-value trend** |
| | 505 (100) | 547 (100) | 603 (100) | | 771 (100.0) | 456 (100.0) | 405 (100.0) | |
| **How did you hear about Coronavirus/COVID-19?** | | | | | | | | |
| Friends or Family | 158 (31.3) | 173 (31.6) | 202 (33.5) | 0.47 | 239 (31.0) | 146 (32.0) | 140 (34.6) | 0.24 |
| Doctor's Office | 79 (15.6) | 90 (16.5) | 105 (17.4) | 0.46 | 132 (17.1) | 66 (14.5) | 70 (17.3) | 0.72 |
| Radio or TV | 250 (49.5) | 305 (55.8) | 396 (65.7) | <.0001 | 449 (58.2) | 251 (55.0) | 239 (59.0) | 0.90 |
| Internet | 320 (63.4) | 399 (72.9) | 422 (70.0) | 0.04 | 525 (68.1) | 306 (67.1) | 294 (72.6) | 0.22 |
| Social Media (Facebook, Twitter etc.) | 341 (67.5) | 393 (71.8) | 392 (65.0) | 0.24 | 507 (65.8) | 311 (68.2) | 295 (72.8) | 0.02 |
| **I am knowledgeable of the risk of Coronavirus** | | | | | | | | |
| Agree | 458 (90.7) | 531 (97.1) | 594 (98.5) | | 739 (95.8) | 440 (96.5) | 384 (94.8) | |
| Disagree | 26 ( 5.1) | 8 ( 1.5) | 2 ( 0.3) | <.0001 | 18 ( 2.3) | 9 ( 2.0) | 9 ( 2.2) | 0.82 |
| **Are you anxious about contracting Coronavirus/COVID-19?** | | | | | | | | |
| Not anxious | 86 (17.0) | 65 (11.9) | 103 (17.1) | | | | | |
| Slightly anxious | 107 (21.2) | 173 (31.6) | 236 (39.1) | | | | | |
| Anxious | 121 (24.0) | 168 (30.7) | 166 (27.5) | | | | | |
| Very anxious | 76 (15.0) | 69 (12.6) | 66 (10.9) | | | | | |
| Really frightened | 100 (19.8) | 65 (11.9) | 29 ( 4.8) | <.0001 | | | | |
| **What worries you most about Coronavirus/COVID-19?** | | | | | | | | |
| Death/fatality | 228 (45.1) | 215 (39.3) | 208 (34.5) | 0.0005 | 228 (29.6) | 199 (43.6) | 219 (54.1) | <.0001 |
| Just the thought of getting the disease | 128 (25.3) | 160 (29.3) | 129 (21.4) | 0.13 | 169 (21.9) | 146 (32.0) | 98 (24.2) | 0.05 |
| Being quarantined | 53 (10.5) | 44 ( 8.0) | 61 (10.1) | 0.95 | 109 (14.1) | 23 ( 5.0) | 24 ( 5.9) | <.0001 |
| Infection/transmission to others | 18 ( 3.6) | 47 ( 8.6) | 89 (14.8) | <.0001 | 91 (11.8) | 39 ( 8.6) | 24 ( 5.9) | <.0001 |
| **If you become ill with Coronavirus/COVID-19, the chances of dying are:** | | | | | | | | |
| Less than 1 in 20 (less than 5%) | 235 (49.0) | 314 (59.6) | 441 (74.4) | | 540 (70.0) | 250 (54.8) | 188 (46.4) | |
| Between 1 in 20 and 1 in 4 (5-25%) | 124 (25.8) | 112 (21.3) | 105 (17.7) | | 122 (15.8) | 123 (27.0) | 95 (23.5) | |
| More than 1 in 4 (More than 25%) | 121 (25.2) | 101 (19.2) | 47 ( 7.9) | <.0001 | 92 (11.9) | 70 (15.4) | 105 (25.9) | <.0001 |

Knowledge is missing for 3 respondents; Anxiety is missing for 26 respondents
